# A HACCP-based approach to mastitis control in dairy herds. Part 1: Development

**DOI:** 10.1186/2046-0481-64-2

**Published:** 2011-03-31

**Authors:** Lies Beekhuis-Gibbon, Paul Whyte, Luke O'Grady, Simon J More, Michael L Doherty

**Affiliations:** 1 School of Agriculture, Food Science and Veterinary Medicine, University College Dublin, Belfield, Dublin 4, Ireland

## Abstract

Hazard Analysis and Critical Control Points (HACCP) systems are a risk based preventive approach developed to increase levels of food safety assurance. This is part 1 of a pilot study on the development, implementation and evaluation of a HACCP-based approach for the control of good udder health in dairy cows. The paper describes the use of a novel approach based on a deconstruction of the infectious process in mastitis to identify Critical Control Points (CCPs) and develop a HACCP-based system to prevent and control mastitis in dairy herds. The approach involved the creation of an Infectious Process Flow Diagram, which was then cross-referenced to two production process flow diagrams of the milking process and cow management cycle. The HACCP plan developed, may be suitable for customisation and implementation on dairy farms. This is a logical, systematic approach to the development of a mastitis control programme that could be used as a template for the development of control programmes for other infectious diseases in the dairy herd.

## Background

Hazard Analysis and Critical Control Points (HACCP) is a preventive risk management approach that has been extensively used by food industries to increase product safety and protect public health [1]. HACCP has been adapted to all stages of the food chain, and is now widely used in dairy and meat processing, and in retail and catering [2,3]. Following the introduction in the European Union of the 'Hygiene Package' in 2004, HACCP-based food safety management systems are now required at all stages of the food chain within the European Union, apart from primary production [4-6].

As yet, agreement has not been reached on robust and practical systems relevant to food safety during primary livestock production. Several papers have examined the potential application of HACCP methods to livestock production [7-9]. However, the recent hygiene package [4], recommends exploration of the feasibility of the application of HACCP during primary production. As an alternative, significant emphasis has been placed on guides to good farming practice (GFP), to encourage the use of appropriate hygiene practices at farm level [4]. These guidelines represent minimum agricultural standards [10], and do not lend themselves to certification, nor do they properly demonstrate the current status of the dairy farm with regard to food safety, animal health and welfare. A more specific approach to managing risks through the application of HACCP at farm level should be more effective in addressing these issues as well as being amenable to certification.

In many European countries, practicing veterinarians have introduced herd health programmes, specifically focusing on animal health and fertility management. Widespread adoption of herd health programmes has been problematic. In a recent United Kingdom (UK) report on veterinary expertise in food animal production, there was a perception among farmers that veterinary input was too focussed on individual animal care, did not 'add value' and was not targeted on profitability, one of the key objectives of optimal herd health implementation [11]. Lievaart et al. [12] highlighted several concerns with herd health programmes, including a lack of structure and clear execution and suggested that HACCP-based programmes could contribute to herd health management, facilitating the delivery of quality control measures for farmers, veterinarians and the consumer.

Milk quality is one of the most important issues facing the dairy industry internationally [13]. Somatic cell counts (SCC) are a key measure of milk quality, reflecting the health status of the mammary gland and the risk of non-physiological changes to milk composition. High SCC has a significant, negative, impact on farm profitability and on milk processing [14]. The five-point mastitis control programme was first devised in the late 1960s [15], and remains the basis for infectious mastitis control. Subsequently, many national mastitis control programmes have been established including those in Australia [16], the Netherlands [17] and the United States [18]. Despite the importance of this issue, and the availability of effective control strategies, in many countries milk quality remains a concern. In a recent review of milk quality internationally and in Ireland, More [14] suggested that the important constraints to national progress towards improved milk quality were problems with effective translation of knowledge to practice, rather than incomplete knowledge *per se*. Furthermore, several authors have highlighted specific problems relating to the effective implementation of mastitis control programmes, including time constraints and insufficient direct economic benefits [19,20] and poor knowledge transfer to farmers [21].

As mentioned previously, concerns have been raised about the feasibility of implementing HACCP systems during primary livestock production. However, there has been limited work examining the application of HACCP systems to milk quality. Therefore, the objective of the present pilot study was to develop, implement and evaluate a HACCP-based approach for the control of mastitis on six Irish dairy farms. This paper (part 1 of the study) will describe the development of a template, based on HACCP principles, suitable for the control of mastitis on dairy farms. The desired output of part 1 of this study was to develop a HACCP-based control programme for mastitis that could be readily customised for use on individual farms.

## Methods

An innovative, HACCP-based approach was used to create a mastitis control programme that could be applied as a disease management tool on individual Irish dairy farms, whilst also enabling external verification.

HACCP principles, as developed by the Codex Alimentarius Commission [22] and further adapted by Noordhuizen et al. [9], were applied to mastitis control in a systematic manner ensuring that all relevant stages, processes or activities taking place on a dairy farm were considered. In brief, this process involves multiple steps, which include assembling a multi-disciplinary team, the creation of a number of process decomposition flow diagrams, identification of hazards and risks and subsequent identification of Critical Control Points (CCPs). For each CCP, appropriate critical limits, monitoring strategies, corrective actions and verification procedures were considered.

The selection of critical control points was carried out initially using a series of flow diagrams developed as part of the study with consideration of the definitions and the decision tree developed previously by the Codex Alimentarius Commission [23] and those of Pierson and Corlett [24]. These state, respectively, that a CCP is 'a step at which control can be applied and is essential to prevent or eliminate a safety hazard or reduce it to an acceptable level' or 'any point in a specific system where loss of control would result in a high probability of a health risk.' It must be acknowledged that the identification of individual steps or processes on farm where hazards and risks can be eliminated or controlled in absolute terms is limited due, for example, to the nature, variation and uncertainty associated with biological systems as encountered on farms. As a result, CCPs met the selection criteria above with the caveat that hazard elimination could not be guaranteed. Noordhuizen et al. [9] suggested the use of Points of Particular Attention (POPA) as an alternative to CCPs for such scenarios while others have recommended the adoption of less rigorous 'type 2' CCPs which require less stringency in terms of outcome/performance as well as, for example, less formal establishment of critical limits and monitoring procedures. In the current study, it was decided to use the formal approach of selecting CCPs and applying them at farm level, while acknowledging the associated uncertainties. It was envisaged that this would enable risks to be reduced to acceptable levels at 'critical' stages through the establishment of objective and measurable critical limits, monitoring procedures, corrective actions and verification procedures. Furthermore, it is expected that the development of a formal HACCP-based system incorporating CCPs will highlight the 'critical' nature of these steps to individual farmers and increase the likelihood that they will focus efforts on these areas and, therefore, increase levels of compliance. Consideration was also given to the fact that such an approach could be more amenable to verification by farm advisors and others.

In the first instance, an interdisciplinary team comprising the five authors, with expertise in veterinary epidemiology, bovine health management and food safety, was assembled. In order to create a HACCP-based system for mastitis control, it was necessary to create two process flow diagrams describing the milking process and the annual cow management cycle, respectively. To identify the potential hazards associated with the key stages in the mastitis infection process, it was also considered necessary to generate a flow diagram representing a deconstruction of the infectious process in mastitis. These flow diagrams were used to identify CCPs by cross-referencing the risk factors for each of the hazards identified in the infectious process flow diagram to the two production process flow diagrams using a colour-coded table (data not shown). Identification of the CCPs was then finalised by cross-referencing these steps with the infection process using the Codex Alimentarius decision tree adapted by Noordhuizen et al. [9]. Subsequently, the establishment of critical limits, monitoring procedures, corrective actions, and verification and documentation procedures were created by reviewing the peer-reviewed scientific literature with a focus on papers dealing with implementation of mastitis control measures [21,25,26] and by drawing on the expertise within the HACCP team.

## Results

### The production process flow diagrams

Two production process decomposition flow diagrams, the Milking Process Flow Diagram and the Cow Management Cycle Flow Diagram, were developed to identify the relevant management activities in the context of mastitis that might occur on an Irish dairy farm. The team considered the potential risks associated with each activity after considering relevant published literature [27-31].

### Milking Process Flow Diagram

**Figure 1:**
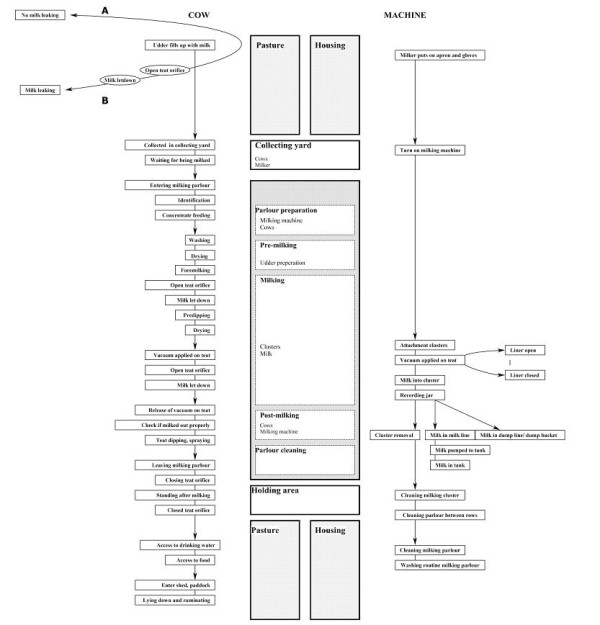
**The milking process flow diagram**.

A Milking Process Flow Diagram (Figure 1) was developed to illustrate the milking process. From left to right, the diagram is divided into three sections relating to cow factors, locations and milking machine factors. The diagram is applicable to two situations. In situation A, the cow does not leak milk before milking, while in situation B, leaking of milk takes place before milking. Both scenarios involve different risks for the incidence in mastitis [32,33].

### Cow Management Cycle Flow Diagram

**Figure 2:**
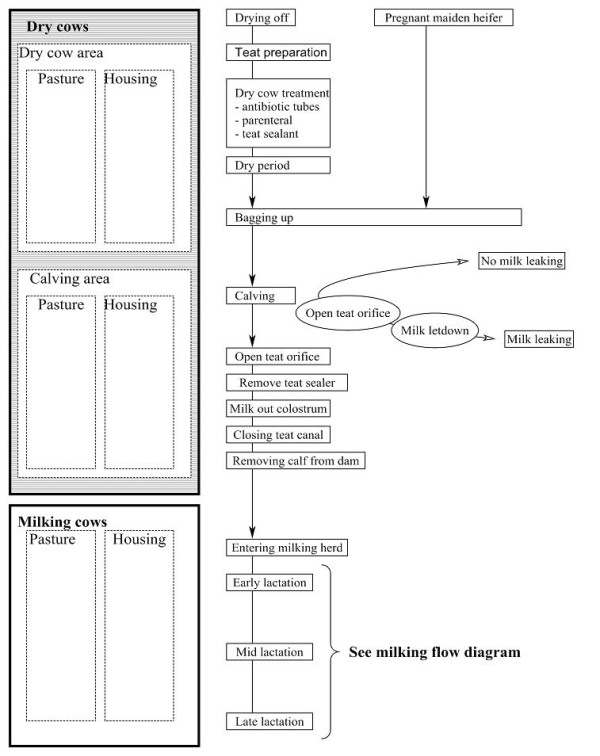
**The cow management cycle flow diagram**.

The Cow Management Cycle Flow Diagram (Figure 2) illustrates the lactation process and is divided into three phases representing the dry period, the calving period and the milking period. The various components relevant to each period are displayed on the right hand side of the diagram. The A and B scenarios are also presented, and the diagram is applicable to both pregnant maiden heifers and cows.

### The Infectious Process Flow Diagram

**Figure 3:**
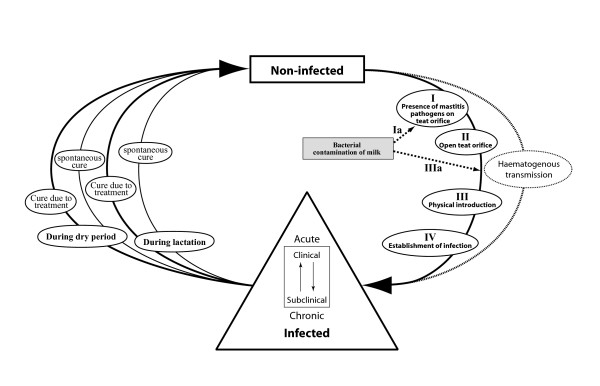
**The infection process flow diagram**.

This conceptual flow diagram (Figure 3) represents a deconstruction of the process of infection occurring in mastitis. The diagram describes the process of infection with mastitis-causing pathogens, up to the clearance of those pathogens. Three steps are critical before establishment of infection (step IV) can take place [34,35].

The critical steps for the establishment of infection are as follows:

I. Presence of mastitis pathogens on the teat orifice.

II. An open teat orifice.

III. Physical introduction of mastitis pathogens.

IV. Establishment of infection.

Before the establishment of infection can occur, mastitis pathogens have to be present on the teat orifice and subsequently introduced to the mammary gland *via *an open teat orifice. When the teat orifice is closed, physical introduction of pathogens is unlikely to take place. The pathway described is consistent for either environmental or contagious mastitis. In both cases, bacteria have to enter the teat canal, and subsequently the parenchyma of the mammary gland, to cause mastitis.

A slight modification of the Infectious Process Flow Diagram takes account of infection due to bacterial contamination of milk that enters/re-enters the teat canal, e.g. in the parlour as follows:

Ia. Bacterial contamination of milk, e.g. due to the presence of mastitis pathogens on the teat orifice.

II. An open teat orifice.

IIIa. Bacterial contamination of milk and physical introduction of mastitis pathogens.

IV Establishment of infection.

A separate pathway was identified for those rare cases in which mastitis develops as a result of haematogenous infection, but was not considered further [36].

When a cow develops mastitis by the mechanisms described, the disease may be clinically detectable or subclinical. The Infectious Process Flow Diagram also represents the shift between clinical and subclinical infection and acute and chronic disease, as well as the various ways in which mastitis can be cured. Cure can take place during lactation or in the dry period, and it can occur spontaneously or as a response to treatment [37].

### Identification of critical control points

The next step in the HACCP-based approach involved the identification of CCPs (see above). To generate these critical control points within the present study, the Infectious Process Flow Diagram (Figure 3) was cross-referenced to the two production process flow diagrams (Figures 1 and 2). Each step identified in the Cow Management Cycle Flow Diagram and Milking Process Flow Diagram was considered in the context of the infection process, to determine if the necessary criteria i.e. the presence of mastitis pathogens on the teat, the teat orifice being open and whether pathogens could be physically introduced, could occur at any of the steps. Specifically, each step identified in the Milking Process Flow Diagram and the Cow Management Cycle Flow Diagram (Figure 1 and 2) was entered in a colour-coded table and assessed in the context of the various mechanisms of exposure; I, Ia, II, III and IIIa (Figure 3). Step IV was not included in this list, because the establishment of infection depends on intrinsic factors, many of which are difficult to influence with most mastitis control measures, e.g. mammary gland immunity and the ability of bacteria to invade mammary tissue [38].

Thereafter, the HACCP team decided when exposure to the various stages of infection could occur. In order to identify CCPs, it was important to ascertain whether stages I, II and III of infection, controlling teat contamination and physical introduction of bacteria could be prevented. When the team decided that exposure at a particular stage could be prevented, the event was highlighted and risk factors for each step(s) were identified based on team expertise and published literature. Six CCPs were identified based on the key principle of preventing physical introduction of mastitis pathogens into the udder and the subsequent establishment of infection, as follows: udder preparation, cluster attachment, post-milking teat disinfection, milking machine monitoring, drying off process, and the calving period.

The determination of hazards, control measures, monitoring strategies, verification methods, corrective actions and critical limits or targets for every identified CCP is fundamental to the HACCP system. After these were determined, all the various components were represented in a table for each CCP (Table 1). Hazards identified by Roman numerals refer back to the various mechanisms of exposure identified in the Infectious Process Flow Diagram (Figure 3).

**Table 1:** Summary of Critical Control Points (CCP) for mastitis control.

CCP	**Hazard**^**1**^	Control measures	Monitoring (Records and Visual inspection)	Verification	Corrective actions (Assessment)
**1**. Udder Preparation	I, Ia II III, IIIa	Washing; Drying; Foremilking; Predipping.	Preparation; Milksocks.	Recent infection rate Total bacterial count Thermoduric count	Udder preparation and cleanliness Milksock records

**2**. Cluster attachment	Ia II	Segregation/Cluster disinfection; Milking machine hygiene; Liner quality.	Cleanliness solution; Frequency of detergent change; Milk recording; Detergent brand; Detergent amount; Machine washing protocol; Liner Quality; Number of milkings/liner.	Recent infection rate; Chronic infection rate; Clinical mastitis rate; Thermoduric count.	Milking management of chronic infected animals; Segregation strategy and recording sheets; Cluster dipping; Milking machine washing protocol; Rubberware care.

**3**. Post milking teat disinfection	II III, IIIa.	Teat disinfection.	Application; Detergent brand; Detergent amount.	Recent infection rate.	Quality and quantity of teat disinfection; Product used.

**4**. Milking machine	I, Ia II III, IIIa	Adequate working milking machine.	Teat end scoring; Assessing liner slippage; Manual vacuum test; Milking machine equipment inspection; Liner change date.	Milking machine report.	Milking machine performance; Teat end scoring.

**5**. Drying off process	I, II, III, IIIa	Teat preparation; Treatment protocol.	Drying off procedure.	Cure rate; New infection rate dry period; Mastitis cases dry cow/heifer.	Teat preparation; Protocol.

**6**. Calving	I, II, III	Hygiene; Shed layout; Stocking.	Visual inspection.	Clinical mastitis cases first 60 days; Recent infection rate first 60 days.	Time spent in area; Pen hygiene; Stocking density.

^1^Hazards are denoted according to potential infection processes illustrated in Figure 3.

Table 1 and other documentation, such as additional in-depth information regarding each CCP, and monitoring sheets were used to create a HACCP-based handbook as previously published [39]. This handbook was then used as a basis for facilitating the development and implementation of HACCP-based mastitis control programme for participating farms. A summary of the contents of the HACCP-based Handbook is presented in Table 2.

**Table 2:** Summary of the contents of the HACCP Implementation Guideline Handbook issued to farmers participating in the study (Beekhuis et al., 2010).

Section	Contents
**1**.	**Introduction to HACCP and Action Research**

**2**.	**Scheduling and information of visits to farms by advisors**

**3**.	**Critical Control Point Information**
	- Udder preparation
	- Cluster attachment
	- Post milking teat disinfection
	- Milking machine
	- Drying off process
	- Calving

**4**.	**Critical Control Point Summary Sheets**
	- Udder preparation
	- Cluster attachment
	- Post milking teat disinfection
	- Milking machine
	- Drying off process
	- Calving

**5**.	**Monitoring and Corrective Action Sheets**
	- Milksock appearance records
	- Segregation group records
	- Teat disinfection use records
	- Milking machine records
	- Clinical mastitis records

**6**.	**Agreed Protocols**

## Discussion

HACCP systems have mainly been applied in the food industry [40], while reports and experiences on the suitability and practicality of applying HACCP systems on farm are variable. Cullor [7] suggested that the HACCP approach was mainly a tool to control food borne and waterborne pathogens. However, evaluation of the applicability of quality control programmes like GFP and HACCP on dairy farms concluded that the HACCP-based approach would yield the best results in the context of animal health, animal welfare and food safety [3]. Noordhuizen et al. [9,41,42] considered that HACCP was a system that could be used as an overall management tool on farm to address all aspects of herd health management. These authors introduced a list of 'General Measures of Prevention' comprising a wide variety of hazards on farm from preventative measures for mastitis, e.g. teat disinfection, up to claw health programmes. Noordhuizen et al. [9,41,42] stated that a wide range of possible hazards would focus attention and increase farmer awareness of herd health. The HACCP-based approaches created by Noordhuizen et al. [9] also identified 'Points of Particular Attention' (POPA) in addition to formal CCPs. However, as many of the POPA were already part of herd health management plans, they do not add significantly to the amount of monitoring and verification required by the system. In the current study, as mentioned previously, it was decided to use the Codex definition for CCP selection in combination with the flow diagrams developed during the study. However, in order to provide sufficient flexibility in our HACCP-based programme on dairy farms, it was acknowledged that CCPs would not necessarily in all instances eliminate risk in a classic HACCP context, but rather control risks to an acceptable level. It was considered that using a more formal approach around the CCP concept would emphasise to individual farmers the need to focus attention on key areas with relevant critical limits, monitoring procedures and corrective actions. In addition, the authors were aware of the need to streamline documentation requirements within the HACCP-based programme so that the administrative burden for farmers and their advisors would be minimised.

A HACCP case study on calf rearing [43] reported positive responses from the farmers involved in terms of animal health benefit, whilst highlighting concerns about establishing CCPs and the time-consuming methodology of the HACCP programme. Gardner [8] reported that HACCP would be inadequate and too costly to use on-farm, as it would require costly diagnostic tests for chemical and drug residues and microbes. Ruegg [44] stated that 'widespread adoption on dairy farms is unlikely because HACCP programmes require critical multidisciplinary review of existing management processes, the establishment of limits via identification of critical control points, the use of routine surveillance procedures, effective record keeping, and documentation of standard processes'.

In the present study, an attempt was made to create a mastitis-focused, streamlined, user-friendly system that would be practical to implement on farm, would minimise on-farm documentation and record keeping but would also facilitate verification. It was also envisaged that the system would also lend itself to further streamlining of monitoring and documentation procedures following implementation at farm level.

Willock et al. [19] and Valeeva et al. [20] reported that farmers were not inclined to change many management factors simultaneously. However, when a preventive approach is focused on performance and the farm-specific mastitis situation, it will result in a more tailored and targeted approach, as mastitis problems on individual farms frequently have different risk factors [45].

The objective of the present study was to ascertain if a HACCP-based approach could be created for potential use as a mastitis control programme. For optimal implementation of the HACCP-based approach developed at farm level, specific tailoring based on an understanding of the mastitis problem on an individual farm will be critical, allowing the opportunity to concentrate on relevant CCPs for each specific farm situation or to specific control measures within CCPs, making the system easier to apply and more adaptable to individual farms. The results of the present study have allowed the creation of a HACCP-based handbook, which can be customised at farm level and facilitate verification (Table 2). The HACCP-based handbook would also provide structure and direction facilitating a 'coaching' role by the veterinarian in the HACCP team and outlining the responsibilities within the partnership of the farmer and veterinarian to tackle mastitis within herds.

While European legislation has not yet made HACCP mandatory for primary production, member states have been prompted to adopt 'HACCP-like' plans to meet the issues of food safety, public health and animal health and welfare [46]. The HACCP-based approach described in the present study offers a logical, structured and formalised approach to mastitis control, which has the potential to be customised for individual dairy farms. The approach developed in this study will be taken to farm-level to assess its practicality and feasibility of implementation [47]. The approach adopted may provide a template for developing a HACCP-based control programme for other infectious diseases of significance to the dairy herd.

## Conclusions

A novel approach based on a deconstruction of the infectious process, the milking process and the cow management cycle was used to develop a HACCP-based system to prevent and control mastitis in dairy herds. The study was the basis for the creation of a HACCP-based handbook, which can be readily modified for specific dairy farms and implemented in collaboration with the veterinary practitioner and farmer. The HACCP-based approach is designed to be user friendly in its implementation, and to lend itself to independent verification with minimal documentation and administrative requirements. The approach adopted may provide a template for developing a HACCP-based control programme for other infectious diseases of significance to the dairy herd.

## Conflict of interest statement

The authors declare that they have no competing interests.

## Authors' contributions

LB-G conducted the literature review and co-ordinated the overall approach to the study. PW provided specific expertise in relation to HACCP principles. LOG, SM and MD provided expertise on bovine health management and epidemiology as it applies to mastitis control. All authors read and approved the final manuscript.
